# The Prognostic Significance of PD1 and PDL1 Gene Expression in Lung Cancer: A Meta-Analysis

**DOI:** 10.3389/fonc.2021.759497

**Published:** 2021-11-18

**Authors:** Chih-Hao Chang, Arthur Chun-Chieh Shih, Ya-Hsuan Chang, Hsuan‐Yu Chen, Ying-Ting Chao, Yi-Chiung Hsu

**Affiliations:** ^1^ Genome and Systems Biology Degree Program, Academia Sinica and National Taiwan University, Taipei, Taiwan; ^2^ Institute of Biomedical Sciences, Academia Sinica, Taipei, Taiwan; ^3^ Institute of Information Science, Academia Sinica, Taipei, Taiwan; ^4^ Institute of Statistical Science, Academia Sinica, Taipei, Taiwan; ^5^ Department of Biomedical Sciences and Engineering, National Central University, Taoyuan, Taiwan

**Keywords:** lung cancer, microarray, immune checkpoint, survival analysis, biomarker

## Abstract

**Background:**

Immune checkpoint blockade therapy represents an extraordinary advance in lung cancer treatment. It is important to determine the expression of immune checkpoint genes, such as programmed cell death 1 (PD1) and programmed cell death-ligand 1 (PDL1), to develop immunotherapeutic strategies. The aim of this study was to explore the association between PD1 and PDL1 gene expression and prognoses and outcomes in lung cancer.

**Methods:**

This meta-analysis analyzed 1,251 patients from eight different microarray gene expression datasets and were evaluated for their prognostic implications and verified using another independent research.

**Results:**

The mean expression levels of PDL1 in adenocarcinoma (AD) and squamous cell carcinoma (SC) were significantly higher in patients who died than in patients who did not. There was a trend toward incremental increases in PD1 and PDL1 expression significantly decreasing the risk of relapse and death among AD patients (HR = 0.69; 95% CI = 0.53 ~ 0.91; HR = 0.68; 95% CI = 0.54 ~ 0.84, respectively) and SC patients (HR = 0.53; 95% CI = 0.32 ~ 0.89; HR = 0.78; 95% CI = 0.57 ~ 1.00 respectively), as early-stage patients in this study were more likely to have high expression of both PD1 and PDL1 than late-stage patients (P-trend < 0.05). In contrast, late-stage SC patients expressing one or more of the genes at a high level had a significantly elevated risk of relapse (HR = 1.51; 95% CI = 1.07 ~ 2.11) and death (HR = 1.41; 95% CI = 1.08 ~ 1.84). This result was consistent with the validation data set.

**Conclusion:**

These findings indicate that high expression of PD1 and PDL1 is associated with superior outcome in early-stage lung cancer but an adverse outcome in late-stage lung cancer. The expression levels of PD1 and PDL1 individually or jointly are potential prognostic factors for predicting patient outcomes in lung cancer.

## Introduction

Lung cancer, especially non-small-cell lung cancer (NSCLC), is the most common cause of cancer-related deaths in the United States and worldwide (1). According to the Cancer Registry Annual Report, 2016, Taiwan (2), the lung cancer age-standardized mortality was 24.02 per hundred thousand and was the highest among the top ten cancer death rates in Taiwan. Approximately 64.46% of lung cancer patients are late-stage NSCLC patients.

Immunotherapy is a new treatment strategy for cancer. The key factor is to strengthen the patient’s immune system to fight the disease (3). Among the many immunotherapeutic strategies, immune checkpoint blockade has numerous advantages in the treatment of many types of cancer. Immune checkpoint blockade enhances antitumor immunity by blocking innate down regulators of immunity, such as cytotoxic T-lymphocyte antigen 4 (CTLA-4) (4) and programmed cell death 1 (PD1) or its ligand, programmed cell death ligand 1 (PDL1) (5). PD1 belongs to the CD28 family and is a coinhibitory surface receptor expressed on activated T cells. It is also expressed on B cells, natural killer (NK) cells, and myeloid-derived suppressor cells (MDSCs) (6–9). PDL1 is the ligand of PD1 and is expressed by antigen-presenting cells and tissue cells, including cancer cells (10–12). The PD1/PDL1 pathway negatively regulates the immune response by inhibiting T cell activation and proliferation, reducing cytokine production, and enhancing CD8 (+) T cell depletion in the tumor microenvironment (13, 14).

A number of studies have described that the expression of the PD1/PDL1 genes is correlated with clinical prognosis. Higher expression of PD1 on CD8+ T cells in tumor tissue was significantly correlated with poor prognosis in pancreatic ductal adenocarcinoma (15), renal cell carcinoma (16) and Hodgkin lymphoma (17). In contrast, tumor tissue infiltration by PD1+ T cells in human papilloma virus (HPV)-associated head and neck cancer (18), follicular lymphoma (19) and colorectal cancer (20) was associated with a good prognosis. For PDL1, high gene expression in renal cell carcinoma (21), urothelial cancers (22), esophageal cancer (23), pancreatic cancer (24), ovarian cancer (25), breast cancer (26) and pulmonary pleomorphic carcinomas (27) was related to a poor clinical outcome. In contrast, stage I pulmonary squamous cell carcinoma (28) and stage I pulmonary adenocarcinoma (29) patients carrying high PDL1 gene expression had a favorable clinical outcome.

Many studies have suggested that PD1/PDL1 play an important role in cancer progression. The association between PD1/PDL1 in different subtypes of cancer, such as pulmonary pleomorphic carcinomas and pulmonary adenocarcinoma or squamous cell carcinoma, or in different clinical cancer stages is still not clear. To further address the relationship between PD1/PDL1 gene expression and different cancer subtypes and the different outcomes associated with different cancer stages, we examined lung cancer microarray datasets with a meta-analysis.

## Materials and Methods

### Construction of Lung Cancer Microarray Database and Covariate Variables

Eight research datasets and one validation cohort GES157011 were collected for verification (**Supplement Table 1**) (30–37) and were restricted to publications with microarray gene expression data and clinical characteristics by using the keywords “Microarray”, “GPL570 (the alternative name of the microarray platform)”, “Lung cancer”, “Clinical information” and “Survival or Relapse status’’ in a search of the Gene Expression Omnibus (GEO, http://www.ncbi.nlm.nih.gov/geo/). The study cohort was combined and stratified by histology, adenocarcinoma (AD) *vs.* squamous cell carcinoma (SC). The variables of interest in this study included type (normal *vs.* tumor tissue), sex (female *vs.* male), smoking history (no *vs.* yes), stage (early: 1 *vs.* late: 2 + 3+4), EGFR mutation status (no *vs.* yes), relapse (no *vs.* yes) and survival status (alive *vs.* dead).

### Adjusting for Batch Effects and Inverse-Variance Weighting

An empirical Bayes method was used to adjust for batch effects in the eight publicly available gene expression datasets (38). The log hazard ratios (HRs) and standard errors (SEs) of the Cox regression model were determined for the fixed effects and the random effects models and for the inverse-variance-weighted method for the meta-analyses. The overall effect determined by the random effects model was significant. Forest plots were constructed to visualize the results. Parameter estimates of all the single studies and the pooled estimates with their confidence intervals were calculated based on the data provided in the plots in **Supplement Figure 1**.

### Statistical Analysis

All statistical analyses were conducted using SAS version 9.4 (Cary, North Carolina). A p-value less than 0.05 was considered statistically significant. The expression levels of PD1 and PDL1 genes and different clinicopathological features were described as the mean ± standard deviation (SD) and the median. The results of statistical analyses were examined against those of an independent t-test for the mean and a Mann-Whitney U test for the median. Receiver operating characteristic (ROC) curves were constructed to calculate an optimal cutoff point to differentiate high or low expression of the PD1 and PDL1 genes. Based on these cutoffs, quantitative measurements of the expression levels of individual genes were converted into binary measurements to help examine whether high or low expression of these genes could be associated with lung cancer progression using progression-free survival (PFS) and overall survival (OS) as outcomes of interest. Survival curves were plotted by using Kaplan–Meier (KM) unadjusted estimation curve analysis. The significance of these associations was assessed using the log-rank test.

The hazard ratio (HR) was reported for the Cox regression model, with corresponding 95% confidence intervals (CIs) and p-values. Because the expression of the two genes was different, several analyses of HRs were performed in the overall histology combined cohort, including the cohort stratified by histology and the cohort analyzing the interaction between stage (early *vs.* late) and histology.

## Result

### The Relationship Between the Expression of the PD1 and PDL1 genes and Clinical Parameters in Lung Cancer

**Figure 1:**
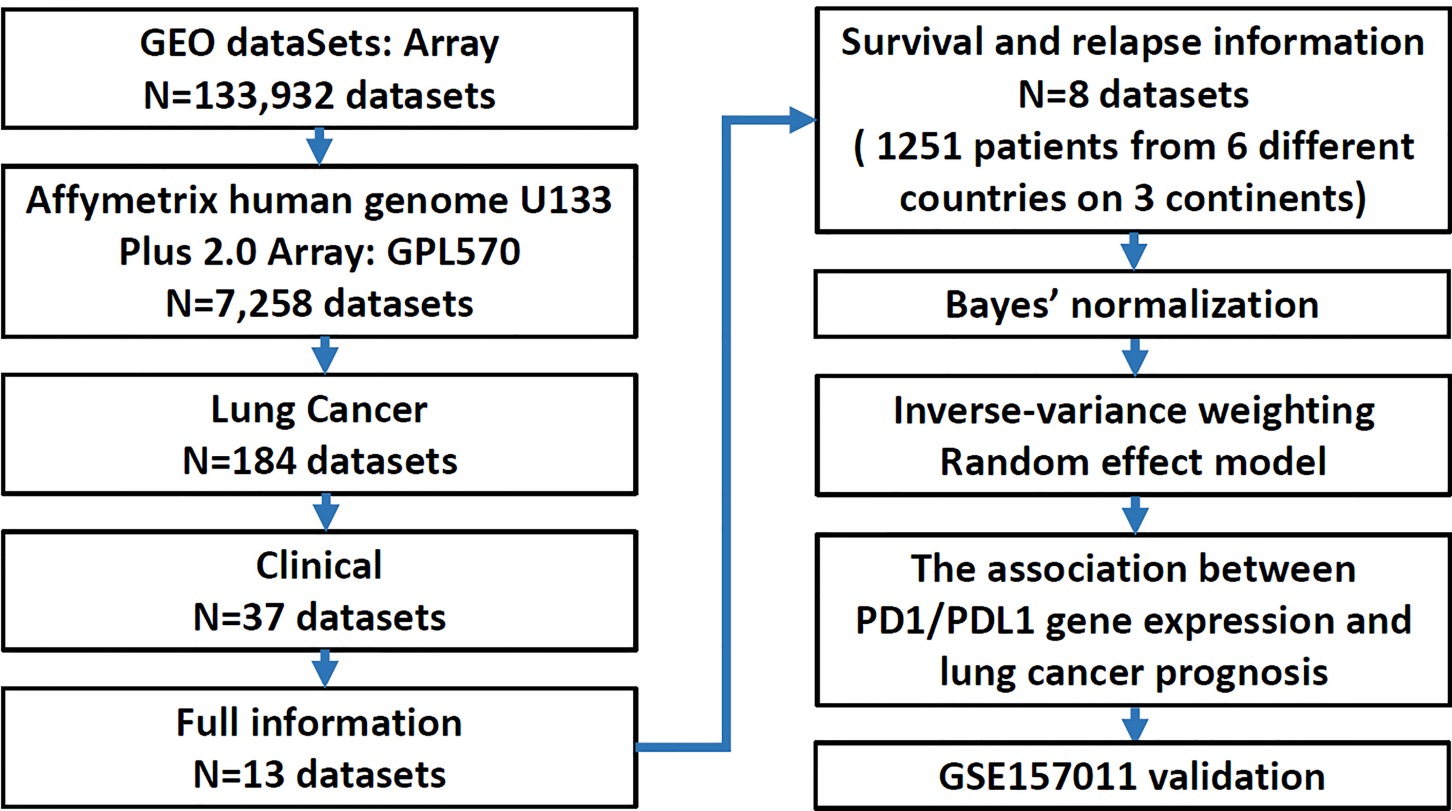
Flow chart summarizing the search process for the identification of eligible datasets.

A total of 1,251 lung cancer patients were studied to delineate the relationship between the expression of PD1 and PDL1 and clinical outcomes in the meta-analysis. A flow diagram of the method used to identify studies to include in the meta-analysis is shown in **Figure 1**. Patients with relapsed lung cancer showed lower expression of these two genes than nonrelapsed patients when histology was combined or divided into adenocarcinoma (AD) and squamous cell carcinoma (SC). Conversely, patients who died of lung cancer demonstrated higher expression of these two genes than patients who did not die of lung cancer. Sex was found to have a significant effect on the expression levels of the two genes (**Table 1**). PD-1 and PDL1 expression were higher in male lung cancer patients than in female lung cancer patients.

**Table 1:** Correlation of PD1 and PDL1 gene expression with clinical parameters of lung cancer patients.

Variable		Adenocarcinoma and Squamous cell carcinoma											
		PD1						PDL1					
		Mean ± SD	Median		P-value^a^	P-value^b^		Mean ± SD	Median			P-value^a^	P-value^b^
**Gender**	**Female**	0.86 ± 0.30	0.86		*Ref.*	*Ref.*		1.03 ± 0.40	1.02			*Ref.*	*Ref.*
	**Male**	0.98 ± 0.42	0.88		**<0.0001**	**0.0349**		1.28 ± 0.56	1.13			**<0.0001**	**<0.0001**
**Stage**	**1**	1.02 ± 0.39	0.94		*Ref.*	*Ref.*		1.21 ± 0.55	1.05			*Ref.*	*Ref.*
	**2+3+4**	0.94 ± 0.37	0.88		**0.0042**	**0.0053**		1.11 ± 0.55	1.05			**0.0188**	0.2115
**Histology**	**Adenocarcinoma**	0.93 ± 0.36	0.90		*Ref.*	*Ref.*		1.13 ± 0.48	1.04			*Ref.*	*Ref.*
	**Squamous cell carcinoma**	0.94 ± 0.44	0.80		0.7980	**0.0034**		1.30 ± 0.58	1.16			**<0.0001**	**<0.0001**
**Relapse**	**No**	0.98 ± 0.39	0.92		*Ref.*	*Ref.*		1.22 ± 0.52	1.09			*Ref.*	*Ref.*
	**Yes**	0.90 ± 0.42	0.84		0.1654	0.0544		1.18 ± 0.55	1.09			0.9614	0.7333
**Survival**	**Alive**	0.96 ± 0.36	0.94		*Ref.*	*Ref.*		1.10 ± 0.51	1.04			*Ref.*	*Ref.*
	**Dead**	1.05 ± 0.40	0.88		**0.0011**	0.6360		1.29 ± 0.59	1.08			**<0.0001**	**0.0074**
		**Adenocarcinoma**											
		**PD1**						**PDL1**					
**Variable**		**Mean ± SD**	**Median**	P-value^a^			P-value^b^	**Mean ± SD**		**Median**	P-value^a^		P-value^b^
**Gender**	**Female**	0.87 ± 0.31	0.87	*Ref.*			*Ref.*	1.03 ± 0.39		1.02	*Ref.*		*Ref.*
	**Male**	0.99 ± 0.39	0.92	**<0.0001**			**0.0028**	1.21 ± 0.53		1.08	**<0.0001**		**<0.0001**
**Stage**	**1**	0.99 ± 0.37	0.94	*Ref.*			*Ref.*	1.15 ± 0.50		1.03	*Ref.*		*Ref.*
	**2+3+4**	0.92 ± 0.32	0.88	**0.0164**			0.0668	1.06 ± 0.49		1.03	**0.0491**		**0.0422**
**Relapse**	**No**	0.97 ± 0.36	0.95	*Ref.*			*Ref.*	1.16 ± 0.47		1.06	*Ref.*		*Ref.*
	**Yes**	0.90 ± 0.39	0.87	**0.0261**			**0.0126**	1.12 ± 0.52		1.06	0.2684		0.6403
**Survival**	**Alive**	0.95 ± 0.34	0.96	*Ref.*			*Ref.*	1.08 ± 0.46		1.03	*Ref.*		*Ref.*
	**Dead**	1.01 ± 0.38	0.87	0.0674			0.3289	1.21 ± 0.55		1.02	**0.0034**		0.5328
		**Squamous cell carcinoma**											
		**PD1**						**PDL1**					
**Variable**		**Mean ± SD**	**Median**	P-value^a^			P-value^b^	**Mean ± SD**		**Median**	P-value^a^		P-value^b^
**Gender**	**Female**	0.84 ± 0.29	0.80	*Ref.*			*Ref.*	1.05 ± 0.46		1.01	*Ref.*		*Ref.*
	**Male**	0.97 ± 0.47	0.80	**0.0085**			0.5275	1.37 ± 0.59		1.22	**<0.0001**		**<0.0001**
**Stage**	**1**	1.13 ± 0.44	0.92	*Ref.*			*Ref.*	1.42 ± 0.66		1.16	*Ref.*		*Ref.*
	**2+3+4**	0.99 ± 0.43	0.85	**0.0211**			**0.0076**	1.21 ± 0.64		1.13	**0.0182**		**0.0490**
**Relapse**	**No**	0.99 ± 0.47	0.82	*Ref.*			*Ref.*	1.38 ± 0.61		1.23	*Ref.*		*Ref.*
	**Yes**	0.91 ± 0.45	0.74	0.1964			0.0567	1.29 ± 0.59		1.22	0.2300		0.3517
**Survival**	**Alive**	0.99 ± 0.46	0.90	*Ref.*			*Ref.*	1.19 ± 0.69		1.08	*Ref.*		*Ref.*
	**Dead**	1.11 ± 0.43	0.90	0.0678			0.1523	1.41 ± 0.63		1.21	**0.0218**		**0.0411**

**SD**, standard deviation; **Ref.**, reference group.

a P value for Independent t-test.

b P value for Mann-Whitney U test.

**Bold face**: statistically significant (P value<0.05).

Among the variables that were significantly different across the histology, the mean (1.30 *vs.* 1.13) and median (1.16 *vs.*1.04) expression levels of PDL1 were higher in SC than in AD (p<0.0001, p<0.0001). The median PD1 expression level was lower in SC than in AD by 0.80 *vs.* 0.90 (p=0.0034). Patients with late-stage disease displayed lower expression of these two genes than those with early-stage disease. There was also a trend for a higher mean expression level of PD1 in SC than in AD, but the difference did not reach statistical significance (**Table 1**).

### Higher Expression of the PD1 and PDL1 genes Acts as a Protective Factor in Lung Cancer Outcomes in Early-Stage Patients

To evaluate the prognostic significance of the expression levels of PD1 and PDL1, we used ROC curves based on the expression levels of PD1 and PDL1 to determine the cutoff values for defining ‘‘high expression’’ and “low expression” (data shown in **Supplement Table 2**). Cox regression analyses and the KM method were used to ascertain the correlations between gene expression and prognostic features.

**Figure 2:**
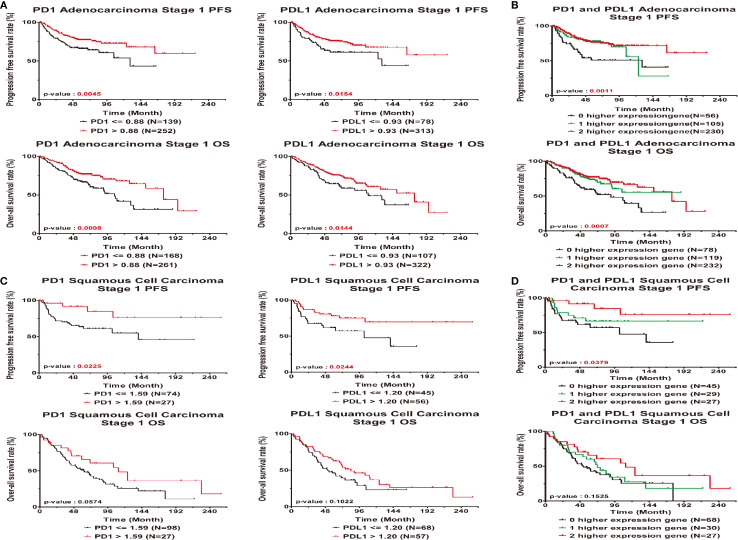
Prognostic correlated with expression levels of PD1 or PDL1 in early stage lung cancer patients. Kaplan-Meier statistical analyses were conducted to examine the association between progression-free survival (top), over-all survival (bottom) and the expression of PD1 and PDL1 in adenocarcinoma **(A)**, squamous cell carcinoma **(C)**, the number of higher expressed genes **(B, D)** in all patients.

Among patients with AD, higher expression of the PD1 and PDL1 genes was a significantly protective factor in terms of relapse and outcomes in early-stage lung cancer patients (P-value for HR<0.05), but the significance of high PDL1 expression as a protective factor was borderline (HR=0.63; 95% CI=0.39 ~ 1.00; P=0.0514). There was a trend toward incremental increases in PD1 and PDL1 expression significantly decreasing the risk for relapse (HR = 0.69; 95% CI = 0.53 ~ 0.91) and for poor outcomes (HR=0.68; 95% CI = 0.54 ~ 0.84), respectively. The higher the expression of the genes was in patients, the lower the ratio of the HR to progression was (P-trend<0.05; **Table 2** left part). KM curves demonstrated that there was a significant difference in the PFS and OS among patients with high PD1 and PDL1 gene expression (**Figure 2A**). There were significant differences in the PFS (log-rank p=0.0011) and OS (log-rank p = 0.0007) among patients without high expression of PD1 or PDL1, patients with high expression of one gene, and patients with high expression of both genes (**Figure 2B**).

**Table 2:** Cox regression analyses of PD1 and PDL1 gene expression levels at early stage lung cancer patients.

	Adenocarcinoma							Squamous Cell Carcinoma				
	Relapse							Relapse				
	No	Yes						No	Yes			
	Number (%)	Number (%)	P-value^a^	HR (95% CI)^b^	P-value			Number (%)	Number (%)	P-value^a^	HR (95% CI)^b^	P-value
**Univariate**							**Univariate**					
**PD1 <= 0.88**	92 (66.19)	47 (33.81)	**0.0228**	***Ref.***			**PD1 <= 1.59**	46 (62.16)	28 (37.84)	**0.0311**	***Ref.***	
**PD1 > 0.88**	195 (77.38)	57 (22.62)		0.62 (0.41 ~ 0.93)	**0.0201**		**PD1 > 1.59**	23 (85.19)	4 (14.81)		0.32 (0.11 ~ 0.96)	**0.0423**
**PDL1 <= 0.93**	49 (62.82)	29 (37.18)	**0.0220**	***Ref.***			**PDL1 <= 1.20**	26 (57.78)	19 (42.22)	0.0534	***Ref.***	
**PDL1 > 0.93**	238 (76.04)	75 (23.96)		0.63 (0.39 ~ 1.00)	0.0514		**PDL1 > 1.20**	43 (76.79)	13 (23.21)		0.45 (0.21 ~ 0.95)	**0.0359**
**Additive model****^c^**				0.69 (0.53 ~ 0.91)	**0.0076**		**Additive model****^c^**				0.53 (0.32 ~ 0.89)	**0.0155**
**Number of higher expression PD1/PDL1**							**Number of higher expression PD1/PDL1**					
**0 higher expression gene**	31 (55.36)	25 (44.64)	**0.0055**	***Ref.***			**0 higher expression gene**	26 (57.78)	19 (42.22)	0.0522	***Ref.***	
**1 higher expression gene**	79 (75.24)	26 (24.76)		0.51 (0.28 ~ 0.92)	**0.0245**		**1 higher expression gene**	20 (68.97)	9 (31.03)		0.61 (0.27 ~ 1.37)	0.2349
**2 higher expression gene**	177 (76.96)	53 (23.04)		0.44 (0.26 ~ 0.75)	**0.0025**		**2 higher expression gene**	23 (85.19)	4 (14.81)		0.26 (0.08 ~ 0.81)	**0.0207**
	**Outcomes**							**Outcomes**				
	**Alive**	**Dead**						**Alive**	**Dead**			
	**Number (%)**	**Number (%)**	**P-value****^a^**	**HR (95% CI)****^b^**		**P-value**		**Number (%)**	**Number (%)**	**P-value****^a^**	**HR (95% CI)****^b^**	**P-value**
**Univariate**							**Univariate**					
**PD1 <= 0.88**	103 (61.31)	65 (38.69)	**0.0074**	***Ref.***			**PD1 <= 1.59**	35 (35.71)	63 (64.29)	0.5017	***Ref.***	
**PD1 > 0.88**	193 (73.95)	68 (26.05)		0.58 (0.41 ~ 0.82)		**0.0023**	**PD1 > 1.59**	12 (44.44)	15 (55.56)		0.63 (0.34 ~ 1.16)	0.1361
**PDL1 <= 0.93**	65 (60.75)	42 (39.25)	**0.0401**	***Ref.***			**PDL1 <= 1.20**	25 (36.76)	43 (63.24)	0.8548	***Ref.***	
**PDL1 > 0.93**	231 (71.74)	91 (28.26)		0.61 (0.42 ~ 0.89)		**0.0101**	**PDL1 > 1.20**	22 (38.60)	35 (61.40)		0.73 (0.45 ~ 1.18)	0.1937
**Additive model** **^c^**				0.68 (0.54 ~ 0.84)		**0.0005**	**Additive model****^c^**				0.78 (0.57 ~ 1.00)	0.0539
**Number of higher expression PD1/PDL1**							**Number of higher expression PD1/PDL1**					
**0 higher expression gene**	42 (53.85)	36 (46.15)	**0.0062**	***Ref.***			**0 higher expression gene**	25 (36.76)	43 (63.24)	0.6839	***Ref.***	
**1 higher expression gene**	84 (70.59)	35 (29.41)		0.49 (0.30 ~ 0.79)		**0.0032**	**1 higher expression gene**	10 (33.33)	20 (66.67)		0.85 (0.49 ~ 1.45)	0.5425
**2 higher expression gene**	170 (73.28)	62 (26.72)		0.43 (0.28 ~ 0.66)		**0.0001**	**2 higher expression gene**	12 (44.44)	15 (55.56)		0.59 (0.31 ~ 1.12)	0.1068

**HR**, Hazard ratio; **CI**, confidence interval; **Ref.**, reference group.

a P-value for Fisher exact test.

b Adjusted for age and gender.

c Trend test for additive model.

**Bold face**: statistically significant (P value<0.05).

Early-stage SC patients with high expression of the PD1 and PDL1 genes were less likely to relapse than early-stage patients without high expression of either gene (HR = 0.32, 95% CI = 0.11 ~ 0.96; p=0.0423 and HR=0.45, 95% CI = 0.21 ~ 0.95; p=0.0359). Among SC patients with high gene expression of both PD1 or PDL1, the risk of relapse was decreased (HR=0.53, 95% CI = 0.32 ~ 0.89; P=0.0155) (**Table 2**). The overall KM curves of PFS illustrated a statistically significant higher survival rate in patients with high expression of a single gene or both genes (log-rank p<0.05) (**Figures 2C, D**), but this relationship was not seen with OS.

### The Correlations Between High Expression of PD1 and PDL1 and Poor Clinical Outcome in Late-Stage Lung Cancer Patients

**Figure 3:**
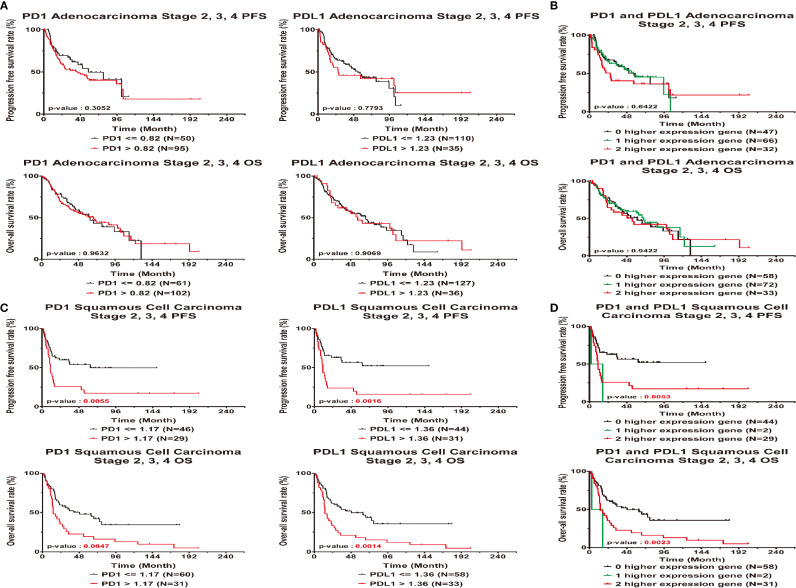
Clinical outcome interacted with expression levels of PD1 or PDL1 in late stage lung cancer patients. Kaplan-Meier statistical analyses were conducted to examine the association between progression-free survival (top), over-all survival (bottom) and the expression of PD1 and PDL1 in adenocarcinoma **(A)**, squamous cell carcinoma **(C)**, the number of higher expressed genes **(B, D)** in all patients.

In addition, high or low PD1 and PDL1 had no significant associations with prognosis or outcome in AD patients. (**Table 3** and **Figures 3A, B**). In contrast, high expression of PD1 and PDL1 was found to be significantly associated with relapse and death in late-stage SC patients, with HRs of 2.08 (95% CI = 1.07 ~ 4.04; p=0.0302) and 2.39 (95% CI = 1.21 ~ 4.69; p=0.0118), respectively, for relapse and 1.87 (95% CI = 1.10 ~ 3.18; p=0.0216) and 2.07 (95% CI = 1.21 ~ 3.53; p=0.0079), respectively, for death. Patients with high expression of both genes were 1.51 times (95% CI = 1.07 ~ 2.11; p=0.0176) more likely to relapse and 1.41 times (95% CI = 1.08 ~ 1.84; p=0.0127) more likely to die (**Table 3**, right part) than patients with high expression of one gene or patients with low expression of both genes.

**Table 3:** Cox regression analyses of PD1 and PDL1 gene expression levels at late stage lung cancer patients.

	Adenocarcinoma							Squamous Cell Carcinoma				
	Relapse							Relapse					
	No	Yes						No	Yes				
	Number (%)	Number (%)	P-value^a^	HR (95% CI)^b^	P-value			Number (%)	Number (%)	P-value^a^	HR (95% CI)^b^	P-value	
**Univariate**							**Univariate**						
**PD1 <= 0.82**	28 (56.00)	22 (44.00)	0.2276	*Ref.*			**PD1 <= 1.17**	25 (54.35)	21 (45.65)	**0.0318**	*Ref.*		
**PD1 > 0.82**	43 (45.26)	52 (54.74)		1.27 (0.75 ~ 2.14)	0.3739		**PD1 > 1.17**	8 (27.59)	21 (72.41)		2.08 (1.07 ~ 4.04)	**0.0302**	
**PDL1 <= 1.23**	57 (51.82)	53 (48.18)	0.2487	*Ref.*			**PDL1 <= 1.36**	25 (56.82)	19 (43.18)	**0.0098**	*Ref.*		
**PDL1 > 1.23**	14 (40.00)	21 (60.00)		1.13 (0.65 ~ 1.96)	0.6772		**PDL1 > 1.36**	8 (25.81)	23 (74.19)		2.39 (1.21 ~ 4.69)	**0.0118**	
**Additive model** **^c^**				1.16 (0.83 ~ 1.63)	0.3932		**Additive model****^c^**				1.51 (1.07 ~ 2.11)	**0.0176**	
**Number of higher expression PD1/PDL1**							**Number of higher expression PD1/PDL1**						
**0 higher expression gene**	25 (53.19)	22 (46.81)	0.1723	*Ref.*			**0 higher expression gene**	25 (56.82)	19 (43.18)	**0.0152**	*Ref.*		
**1 higher expression gene**	35 (53.03)	31 (46.97)		0.97 (0.55 ~ 1.71)	0.9174		**1 higher expression gene**	0 (0.00)	2 (100.00)		3.73 (0.83 ~ 16.84)	0.0865	
**2 higher expression gene**	11 (34.38)	21 (65.63)		1.37 (0.71 ~ 2.65)	0.3548		**2 higher expression gene**	8 (27.59)	21 (72.41)		2.31 (1.16 ~ 4.60)	**0.0170**	
	**Outcomes**							**Outcomes**					
	**Alive**	**Dead**						**Alive**	**Dead**				
	**Number (%)**	**Number (%)**	**P-value****^a^**	**HR (95% CI)****^b^**	**P-value**			**Number (%)**	**Number (%)**	**P-value****^a^**	**HR (95% CI)****^b^**	**P-value**	
**Univariate**							**Univariate**						
**PD1 <= 0.82**	29 (47.54)	32 (52.46)	0.8725	*Ref.*			**PD1 <= 1.17**	26 (43.33)	34 (56.67)	**0.0002**	*Ref.*		
**PD1 > 0.82**	47 (46.08)	55 (53.92)		0.89 (0.57 ~ 1.40)	0.6177		**PD1 > 1.17**	2 (6.45)	29 (93.55)		1.87 (1.10 ~ 3.18)	**0.0216**	
**PDL1 <= 1.23**	62 (48.82)	65 (51.18)	0.3459	*Ref.*			**PDL1 <= 1.36**	26 (44.83)	32 (55.17)	**0.0001**	*Ref.*		
**PDL1 > 1.23**	14 (38.89)	22 (61.11)		0.90 (0.53 ~ 1.51)	0.6829		**PDL1 > 1.36**	2 (6.06)	31 (93.94)		2.07 (1.21 ~ 3.53)	**0.0079**	
**Additive model** **^c^**				0.92 (0.68 ~ 1.24)	0.5681		**Additive model****^c^**				1.41 (1.08 ~ 1.84)	**0.0127**	
**Number of higher expression PD1/PDL1**							**Number of higher expression PD1/PDL1**						
**0 higher expression gene**	27 (46.55)	31 (53.45)	**0.3607**	***Ref.***			**0 higher expression gene**	26 (44.83)	32 (55.17)	**0.0001**	*Ref.*		
**1 higher expression gene**	37 (51.39)	35 (48.61)		0.82 (0.51 ~ 1.35)	0.4395		**1 higher expression gene**	0 (0.00)	2 (100.00)		4.68 (1.07 ~ 20.38)	**0.0400**	
**2 higher expression gene**	12 (36.36)	21 (63.64)		0.86 (0.48 ~ 1.57)	0.6321		**2 higher expression gene**	2 (6.45)	29 (93.55)		2.00 (1.16 ~ 3.44)	**0.0126**	

**HR**, Hazard ratio; **CI**, confidence interval; **Ref.**, reference group.

a P-value for Fisher exact test.

b Adjusted for age and gender.

c Trend test for additive model.

**Bold face**: statistically significant (P value<0.05).

Based on the KM curves, high and low PD1 and PDL1 expression levels were significantly associated with PFS and OS (**Figures 3C, D**) in squamous cell carcinoma. High gene expression was associated with a worse survival rate than low gene expression (log-rank p<0.05). There were significant differences in the PFS (log-rank p=0.0053) and OS (log-rank p = 0.0023) between patients with low expression of both genes, patients with high expression of one gene and patients with high expression of both genes.

### Verify the Relationship Between PD1 and PDL1 Gene Expression and Clinical Prognosis

**Figure 4:**
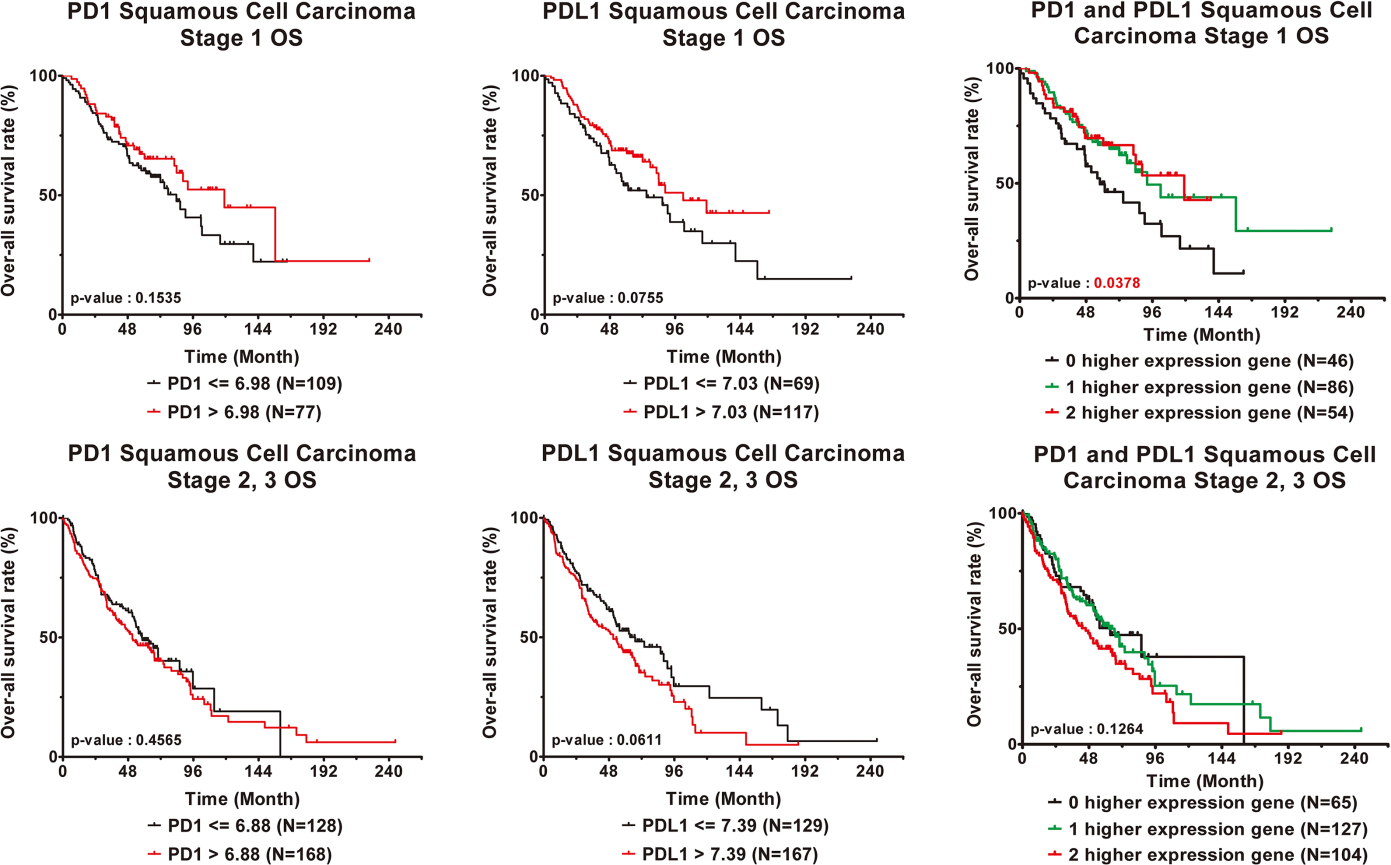
Clinical outcome interacted with expression levels of PD1 or PDL1 in squamous cell carcinoma lung cancer patients. Kaplan-Meier statistical analyses were conducted to examine the association between over-all survival and the expression of PD1 and PDL1 and the number of higher expressed genes in early stage (top) and late stage (bottom) squamous cell carcinoma lung cancer patients.

The independent validation cohort GSE157011 was used to analyze the relationship between PD1/PDL1 gene expression and clinical prognosis in patients with lung squamous cell carcinoma. **Supplement Table 1** contains the clinical characteristics. The PD1 gene expression level of patients with advanced lung squamous cell carcinoma is lower than those of early stage patients (**Table 4**). According to the correlation analysis between PD1/PDL1 gene expression and clinical prognosis (**Table 5**) and survival analysis (**Figure 4**), the higher PD1/PDL1 gene expression in patients with early stage lung squamous cell carcinoma had a better prognosis. On the contrary, the prognoses of patients with higher gene expression were worse in the late stage patients.

**Table 4:** Correlation of PD1 and PDL1 gene expression with clinical parameters of squamous cell carcinoma lung cancer patients in the validation dataset.

		PD1				PDL1			
Variable		Mean ± SD	Median	P-value^a^	P-value^b^	Mean ± SD	Median	P-value^a^	P-value^b^
**Gender**	**Female**	6.94 ± 0.12	6.93	***Ref.***	***Ref.***	7.43 ± 0.66	7.21	***Ref.***	***Ref.***
	**Male**	6.93 ± 0.12	6.91	0.5671	0.4460	7.43 ± 0.67	7.23	0.9884	0.7380
**Stage**	**1**	6.96 ± 0.12	6.95	***Ref.***	***Ref.***	7.34 ± 0.59	7.18	***Ref.***	***Ref.***
	**2+3**	6.92 ± 0.12	6.91	**0.0003**	**0.0004**	7.48 ± 0.71	7.28	**0.0205**	0.0736
**Survival**	**Alive**	6.93 ± 0.13	6.92	***Ref.***	***Ref.***	7.48 ± 0.69	7.26	***Ref.***	***Ref.***
	**Dead**	6.93 ± 0.12	6.92	0.8604	0.9513	7.38 ± 0.64	7.21	0.0988	0.0857

**SD**, standard deviation; **Ref.**, reference group.

a P value for Independent t-test.

b P value for Mann-Whitney U test.

**Bold face**: statistically significant (P value<0.05).

**Table 5:** Cox regression analyses of PD1 and PDL1 gene expression levels in early and late stage squamous cell carcinoma lung cancer patients.

Early stage						
	Outcomes			HR (95% CI)^b^		P-value
	Alive	Dead				
	Number (%)	Number (%)	P-value^a^			
**Univariate**						
**PD1 <= 6.98**	55 (50.46)	54 (49.54)	0.1791	***Ref.***		
**PD1 > 6.98**	47 (61.04)	30 (38.96)		0.72 (0.46 ~ 1.13)		0.1552
**PDL1 <= 7.03**	30 (43.48)	39 (56.52)	**0.0220**	***Ref.***		
**PDL1 > 7.03**	72 (61.54)	45 (38.46)		0.68 (0.44 ~ 1.04)		0.0773
Additive model^c^				0.72 (0.53 ~ 0.97)		**0.0300**
**Number of higher expression PD1/PDL1**			**0.0175**			
**0 higher expression gene**	17 (36.96)	29 (63.04)		***Ref.***		
**1 higher expression gene**	51 (59.30)	35 (40.70)		0.57 (0.35 ~ 0.94)		**0.0267**
**2 higher expression gene**	34 (62.96)	20 (37.04)		0.54 (0.31 ~ 0.96)		**0.0347**
**Late stage**						
	**Outcomes**					
	**Alive**	**Dead**				
	**Number (%)**	**Number (%)**	**P-value****^a^**	**HR (95% CI)****^b^**		**P-value**
**Univariate**						
**PD1 <= 6.88**	58 (45.31)	70 (54.69)	0.0929	***Ref.***		
**PD1 > 6.88**	59 (35.12)	109 (64.88)		1.12 (0.83 ~ 1.52)		0.4569
**PDL1 <= 7.39**	59 (45.74)	70 (54.26)	0.0566	***Ref.***		
**PDL1 > 7.39**	58 (34.73)	45 (65.27)		1.33 (0.99 ~ 1.80)		0.0621
Additive model^c^				1.21 (0.98 ~ 1.48)		0.0747
**Number of higher expression PD1/PDL1**			**0.0461**			
**0 higher expression gene**	32 (49.23)	33 (50.77)		***Ref.***		
**1 higher expression gene**	53 (41.73)	74 (58.27)		1.02 (0.68 ~ 1.54)		0.9222
**2 higher expression gene**	32 (30.77)	72 (69.23)		1.38 (0.92 ~ 2.09)		0.1242

**HR**: Hazard ratio, **CI**: confidence interval, **Ref.**: reference group

a P-value for Fisher exact test.

b Adjusted for age and gender.

c Trend test for additive model.

**Bold face**: statistically significant (P value<0.05).

## Discussion

Lung cancer is the major cause of mortality worldwide (1). This study supports that PD1/PDL1 gene expression is a predictor of lung cancer prognosis in early- and late-stage NSCLC.

We examined the relationship between PD1/PDL1 and clinical outcomes in the different cancer subtypes. All analyses were meta-analyses, and to minimalize the differences between the eight collated microarray databases, an empirical Bayes method was used to adjust for batch effects in the eight publicly available gene expression datasets (38).

The Bayes normalization method is robust for adjusting for batch effects in studies in which the batch sizes are small. This method was designed to stabilize very high or very low gene expression levels by dampening the variability across all other genes. We used the empirical Bayes method in this study instead of the quantile normalization method (39) because we found that the estimate of heterogeneity between different datasets calculated with the Bayes normalization method was better than that found with the quantile normalization method by mean or by median (40) (**Supplement Table 3** and **Figure 2**).

The PD1/PDL1 axis is a checkpoint in immune cells. It normally acts as a type of “off switch” that helps keep T cells from attacking other cells in the body. When PD1 attaches to PDL1, it directs the T cell to ignore the other cell. PD1/PDL1 immune checkpoint blockade mechanisms inhibit this binding and boost the immune response against cancer cells. The PD1 inhibitors, nivolumab and pembrolizumab, and the PDL1 inhibitors, atezolizumab, avelumab and durvalumab (41), unleash antitumor immunity to achieve therapeutic effects (3).

The findings of this study agree with the results (28, 29) that higher PD1/PDL1 gene expression had better prognosis in the early stage among AD and SC lung cancer patients. Furthermore, we observed that higher PD1/PDL1 gene expression turned into the risk factors for worse clinical outcomes among the late stage AD and SC lung cancer patients. When exploring the relationship between higher gene number and prognosis or outcomes, we found that for most, but not all, the higher gene expression and higher number of expression genes are more likely to bind together, therefore, the blocking mechanism avoids triggering the anti-tumor immunity, which causes the cancer cell to remain active in the body.

The limitation of this study was that only eight microarray databases were available for the search strategy. Nevertheless, the study was statistically well adjusted. Our meta-analysis of eight different lung cancer studies demonstrates the impact of PD1/PDL1 gene expression on NSCLC prognosis. Importantly, our analyses indicate that late-stage NSCLC patients with high expression of PD1 and PDL1, either individually or jointly, tend to suffer a greater risk of recurrence or death than patients with early-stage NSCLC. Conversely, in early-stage NSCLC patients, high gene expression is associated with a favorable clinical outcome. Therefore, our results support that PD1 and PDL1 are valuable markers for the prognostication of NSCLC.

## Data Availability Statement

The original contributions presented in the study are included in the article/**Supplementary Material**. Further inquiries can be directed to the corresponding author.

## Author Contributions

H-YC, Y-CH, and C-HC made substantial contributions to conception and design. C-HC, Y-CH, and Y-TC wrote the manuscript. C-HC interpreted the data and analyzed the data with AC-CS and Y-HC. All authors approved the final manuscript.

## Funding

This research was supported by Academia Sinica and the Ministry of Science and Technology (AS-104-TP-A07, MOST 104-0210-01-09-02, MOST 105-0210-01-13-01, MOST 106-0210-01-15-02, and MOST 107-2314-B-008-002, and MOST 110-2221-E-008-048) and the Next-Generation Pathway of Taiwan Cancer Precision Medicine Program (AS-KPQ-107-TCPMP).

## Conflict of Interest

The authors declare that the research was conducted in the absence of any commercial or financial relationships that could be construed as a potential conflict of interest.

## Publisher’s Note

All claims expressed in this article are solely those of the authors and do not necessarily represent those of their affiliated organizations, or those of the publisher, the editors and the reviewers. Any product that may be evaluated in this article, or claim that may be made by its manufacturer, is not guaranteed or endorsed by the publisher.
