# Supplementary material for: The Prognostic Significance of PD1 and PDL1 Gene Expression in Lung Cancer: A Meta-Analysis
Source: Front Oncol. 2021 Nov 18;11:759497. doi: 10.3389/fonc.2021.759497 (PMC8639141; doi:10.3389/fonc.2021.759497)
Supplement: Supplementary file 1 [file DataSheet_1.pdf]

Supplement Table 1 Clinical characteristics of lung cancer patients

		Research									Validate
		Total	GSE31210	GSE8894	GSE30219	GSE37745	GSE50081	GSE3141	GSE31908	GSE29013	GSE157011
**Study**		**Number (%)**	**Number (%)**	**Number (%)**	**Number (%)**	**Number (%)**	**Number (%)**	**Number (%)**	**Number (%)**	**Number (%)**	**Number (%)**
**Country**			Japan	South Korea	France	Sweden	Canada	USA	USA	USA	USA
**Sample Size**		1251 (100.00)	246 (19.66)	138 (11.03)	307 (24.54)	196 (15.67)	181 (14.16)	111 (8.87)	17 (1.36)	55 (4.40)	482 (100.00)
**Age**	**Mean ± SD (range)**	62.68 ± 10.17 (13 ~ 89)	59.54 ± 8.08 (30 ~ 89)	60.78 ± 9.58 (13 ~ 82)	61.51 ± 11.58 (15 ~ 84)	63.77 ± 9.18 (39 ~ 84)	68.79 ± 9.4 (40 ~ 88)			63.58 ± 8.73 (32 ~ 76)	68.94 ± 8.63 (43 ~ 92)
**Type**	**Normal**	35 (2.80)	20 (8.13)		14 (4.56)				1 (5.88)		
	**Tumor**	1216 (97.20)	226 (91.87)	138 (100.00)	293 (95.44)	196 (100.00)	181 (100.00)	111 (100.00)	16 (94.12)	55 (0.00)	482 (100.00)
**Gender**	**Male**	716 (63.59)	116 (47.15)	104 (75.36)	250 (85.32)	107 (54.59)	98 (54.14)		3 (17.65)	38 (69.09)	313 (64.94)
	**Female**	410 (36.41)	130 (52.85)	34 (24.64)	43 (14.68)	89 (45.41)	83 (45.86)		14 (82.35)	17 (30.91)	169 (35.06)
**Smoking**	**No**	153 (32.08)	123 (50.00)				24 (15.00)		4 (25.00)	2 (3.64)	
	**Yes**	324 (67.92)	123 (50.00)				136 (85.00)		12 (75.00)	53 (96.36)	
**Stage**	**1**	612 (63.29)	168 (74.34)		152 (51.88)	130 (66.33)	127 (70.17)		11 (68.75)	24 (43.64)	186 (38.59)
	**2**	209 (21.61)	58 (25.66)		43 (14.68)	35 (17.86)	54 (29.83)		5 (31.25)	14 (25.45)	247 (51.24)
	**3**	131 (13.55)			87 (29.69)	27 (13.78)				17 (30.91)	49 (10.17)
	**4**	15 (1.55)			11 (3.75)	4 (2.04)					
**EGFR mutation**	**No**	107 (44.21)	99 (43.81)						8 (50.00)		
	**Yes**	135 (55.79)	127 (56.19)						8 (50.00)		
**Histology**	**Adenocarcinoma**	713 (57.97)	226 (100.00)	63 (45.65)	85 (27.69)	106 (54.08)	129 (71.27)	58 (52.25)	16 (100.00)	30 (54.55)	
	**Squamous cell carcinoma**	344 (27.97)		75 (54.35)	82 (26.71)	66 (33.67)	43 (23.76)	53 (47.75)		25 (45.45)	482 (100.00)
	**Large cell carcinoma**	92 (7.48)			59 (19.22)	24 (12.24)	9 (4.97)				
	**Others**	81 (6.59)			81 (26.38)						
**Relapse**	**No**	603 (61.16)	162 (71.68)	69 (50.00)	164 (58.99)	47 (48.96)	126 (71.19)		8 (50.00)	27 (49.09)	
	**Yes**	383 (38.84)	64 (28.32)	69 (50.00)	114 (41.01)	49 (51.04)	51 (28.81)		8 (50.00)	28 (50.91)	
**Survival**	**Alive**	545 (50.56)	191 (84.51)		93 (31.74)	51 (26.02)	106 (58.56)	53 (47.75)	14 (87.50)	37 (62.27)	219 (45.44)
	**Dead**	533 (49.44)	35 (15.49)		200 (68.26)	145 (73.98)	75 (41.44)	58 (52.25)	2 (12.50)	18 (32.73)	263 (54.56)

SD: standard deviation

Supplement Table 2 ROC analysis of gene expression of PD1 and PDL1 for predicting outcome in lung cancer patients

	Gene expression			
	PD1		PDL1	
	AUC (95%CI)	P-value [a]	AUC (95%CI)	P-value [a]
**Non-relapse vs. relapse**				
**Adenocarcinoma stage 1**	0.55 (0.48 ~ 0.62)	0.1377	0.54 (0.48 ~ 0.61)	0.2129
**Adenocarcinoma stage 2+3+4**	0.55 (0.46 ~ 0.65)	0.2565	0.51 (0.42 ~ 0.61)	0.7681
**Squamous cell carcinoma stage 1**	0.60 (0.49 ~ 0.72)	0.0839	0.59 (0.47 ~ 0.72)	0.1456
**Squamous cell carcinoma stage 2+3+4**	0.57 (0.43 ~ 0.70)	0.3272	0.59 (0.46 ~ 0.73)	0.1603
**Alive vs. death**				
**Adenocarcinoma stage 1**	0.72 (0.67 ~ 0.78)	**<0.0001**	0.74 (0.69 ~ 0.79)	**<0.0001**
**Adenocarcinoma stage 2+3+4**	0.77 (0.69 ~ 0.84)	**<0.0001**	0.77 (0.69 ~ 0.84)	**<0.0001**
**Squamous cell carcinoma stage 1**	0.55 (0.44 ~ 0.67)	0.3845	0.59 (0.48 ~ 0.70)	0.0964
**Squamous cell carcinoma stage 2+3+4**	0.79 (0.68 ~ 0.89)	**<0.0001**	0.78 (0.67 ~ 0.89)	**<0.0001**

ROC: receiver operating characteristic, **AUC**: areas under the ROC curves, **CI**: confidence interval

[a]: P value for Mann-Whitney U test

Bold face: statistical significant (P value<0.05)

Supplement Table 3 PD1 and PDL1 gene Affymetrix probes gene expression at different study

	PD1							
	Raw data		Quantile normalization by mean		Quantile normalization by Median		Bayes' normalization	
	Mean ± SD	P-Value [a]	Mean ± SD	P-Value [a]	Mean ± SD	P-Value [a]	Mean ± SD	P-Value [a]
		<0.0001		<0.0001		<0.0001		**0.9377**
**GSE50081**	5.37 ± 0.35		66.18 ± 20.51		6.03 ± 0.27		5.32 ± 0.64	
**GSE30219**	5.69 ± 0.23		64.51 ± 10.6		6.02 ± 0.15		5.32 ± 0.63	
**GSE31210**	34.76 ± 20.69		15.21 ± 7.89		4.17 ± 0.64		5.32 ± 0.66	
**GSE8894**	2.33 ± 0.16		7.51 ± 7.05		3.49 ± 0.48		5.32 ± 0.58	
**GSE37745**	6.80 ± 0.24		79.01 ± 14.72		6.22 ± 0.19		5.32 ± 0.62	
**GSE3141**	102.39 ± 91.41		18.46 ± 15.31		4.27 ± 0.83		5.31 ± 0.66	
**GSE31908**	6.25 ± 0.20		47.32 ± 8.2		5.72 ± 0.19		5.32 ± 0.58	
**GSE29013**	5.34 ± 1.08		45.85 ± 54.47		5.11 ± 1.10		5.32 ± 0.65	

	PDL1							
	Raw data		Quantile normalization by mean		Quantile normalization by Median		Bayes' normalization	
	Mean ± SD	P-Value [a]	Mean ± SD	P-Value [a]	Mean ± SD	P-Value [a]	Mean ± SD	P-Value [a]
		<0.0001		<0.0001		**0.1297**		**0.3560**
**GSE50081**	5.35 ± 0.78		92.28 ± 111.50		6.06 ± 0.70		6.67 ± 0.93	
**GSE30219**	5.92 ± 1.02		120.52 ± 155.33		6.26 ± 0.95		6.67 ± 0.97	
**GSE31210**	628.08 ± 429.63		180.15 ± 112.16		6.82 ± 0.56		6.67 ± 0.89	
**GSE8894**	4.26 ± 1.45		150.85 ± 207.27		6.10 ± 1.21		6.67 ± 0.95	
**GSE37745**	7.22 ± 0.97		175.99 ± 193.27		6.73 ± 0.96		6.67 ± 0.96	
**GSE3141**	689.58 ± 508.63		193.57 ± 174.07		6.92 ± 0.89		6.67 ± 0.84	
**GSE31908**	6.47 ± 0.28		75.46 ± 24.18		5.90 ± 0.33		6.66 ± 0.76	
**GSE29013**	6.97 ± 0.75		222.37 ± 182.97		7.17 ± 1.10		6.67 ± 0.70	

SD: standard deviation

[a]: P-value for homogeneity Levene's Test

Bold face: statistical significant (P value > 0.05)

Supplement Figure 1 PD1 and PDL1 gene expression forest plot

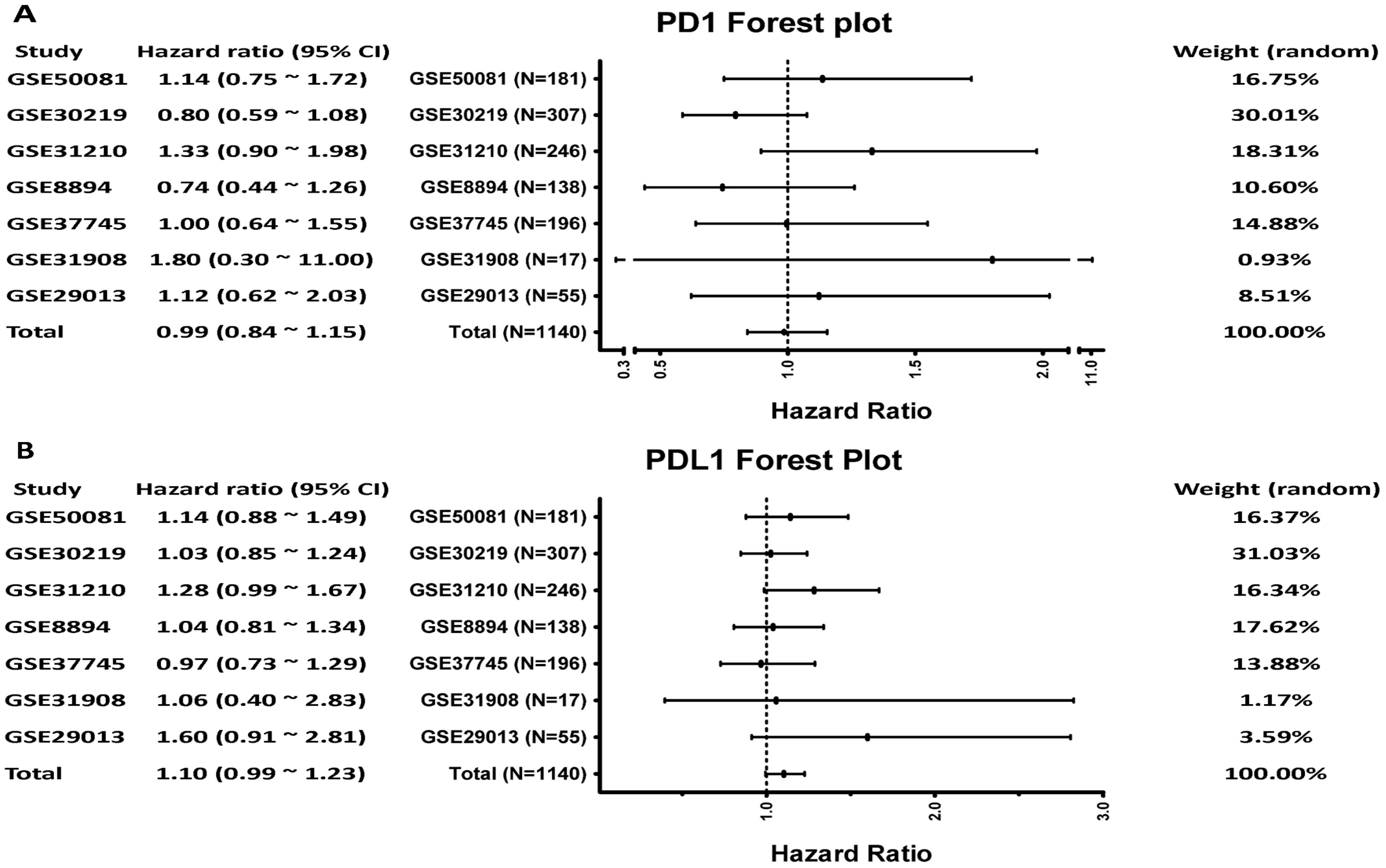

Meta-analyses random effects model forest plot in PD1 (A), PDL1 (B).

Supplement Figure 2 PD1 and PDL1 gene expression in different normalized methods

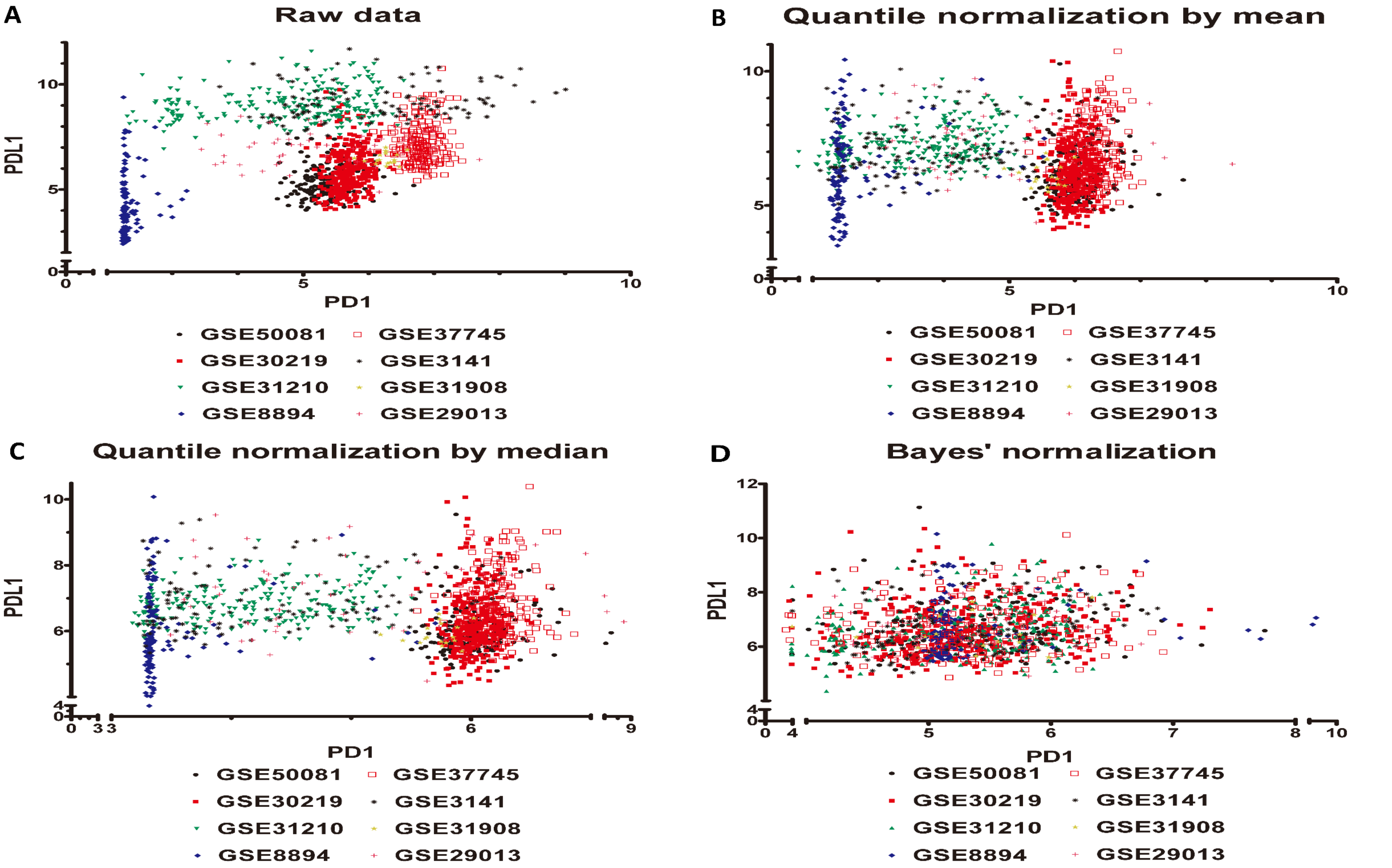

Scatter plot of PD1 and PDL1 gene expression in raw data(A), quantile normalization by mean (B), quantile normalization by median (C), and Bayes' normalization (D).
